# Zoonotic helminths parasites in the digestive tract of feral dogs and cats in Guangxi, China

**DOI:** 10.1186/s12917-015-0521-7

**Published:** 2015-08-16

**Authors:** Fang Fang, Jian Li, Tengfei Huang, Jacques Guillot, Weiyi Huang

**Affiliations:** Laboratory of parasitology, College of Animal Science and Technology, Guangxi University, Nanning, 530004 Guangxi China; Food Quality and Safety Center, Guangxi University, Nanning, Guangxi China; Research group ENVA, UPEC Dynamyc, Ecole Nationale Vétérinaire d’Alfort, Maisons-Alfort, UPE France

**Keywords:** Dogs, Cats, Helminths, Zoonosis, Guangxi, China

## Abstract

**Background:**

In Guangxi, a province of southern China, an important number of dogs and cats roam freely in rural settings, and the presence of these animals in proximity of people may represent a risk of parasitic zoonoses. The objective of the present study was to investigate the presence and identify gastrointestinal helminths in feral carnivores in Guangxi province. Therefore, post mortem examination was performed in 40 dogs and in 39 cats.

**Results:**

The Gastrointestinal helminths were found in all the necropsied dogs and in 37 out of 39 cats. Fifteen species were identified including 7 trematodes, 3 cestodes and 5 nematodes. Most of them may be responsible for zoonotic infections.

**Conclusions:**

Major zoonotic gastrointestinal helminths, including liver and intestinal flukes, *Toxocara* spp., and *Ancylostoma* spp., are present in feral dogs and cats in Guangxi, and may represent a significant risk for public health.

## Background

The potential role of carnivores as reservoirs for zoonotic pathogens has been recognized as a significant public health concern worldwide [1–3]. Among zoonotic agents, helminths of dogs and cats constitute a diversified group including trematodes, cestodes and nematodes. Carnivores usually act as definitive hosts and they contribute to the transmission of zoonotic infections by disseminating infective eggs or larvae in the environment (in the case of *Toxocara* spp., *Ancylostoma* spp., *Strongyloides stercoralis* or *Echinococcus* spp.) or by contaminating intermediate hosts that may be further consumed by humans (especially in the case of fishborne zoonotic trematodes like *Clonorchis sinensis*). Moreover, in some Chinese regions, dogs and cats may be eaten and represent a source of human infection by *Trichinella* spp. In a recent review, Chen et al. mentioned that canine and feline trichinellosis was reported in 11 and 10 endemic Chineses provinces, respectively [2].

In Southern China, a large number of dogs and cats roam freely in rural settings and the presence of these animals in proximity with people may represent a risk of parasitic zoonoses. The objective of the present study was to investigate the presence and identify gastrointestinal helminths in feral dogs and cats in Guangxi province.

## Methods

Gastrointestinal helminths were identified in 40 dogs and 39 cats. The study was conducted from March to October 2012. All of the dogs were collected from Binyang county (near the city of Nanning) and examined at Guangxi Zhuang autonomous region center for disease control and prevention. All of the cats were bought from markets in Nanning. Based on the legislation to protect the welfare of animals and taking into consideration the 3Rs, the animals were sacrificed according to the agreed policy and principles for animal euthanasia and following the guidelines of the Ethical Committee of Guangxi University, which provided a formal approval to the study. The liver, stomach, small and large intestine were separated into four Petri dishes. Each of the portions was cut longitudinally and only the larger parasites were collected. The remaining content was collected in a sediment cup. After several cycles of sedimentation and suspension, the final sediment was poured into a Petri dish and examined under a stereoscopic microscope. All the gastrointestinal helminths were collected and fixed in 70 % alcohol or 10 % formalin. The number of individuals of each species was recorded. Cestodes count was based on the number of scoleces. For further identification, the trematodes and the cestodes were flatten and stained with hydrochloric acid carmine. The nematodes were cleared with lactophenol. All the parasites (trematodes, cestodes and nematodes) were identified morphologically to species according to identification keys [3–6].

## Results and discussion

**Fig. 1:**
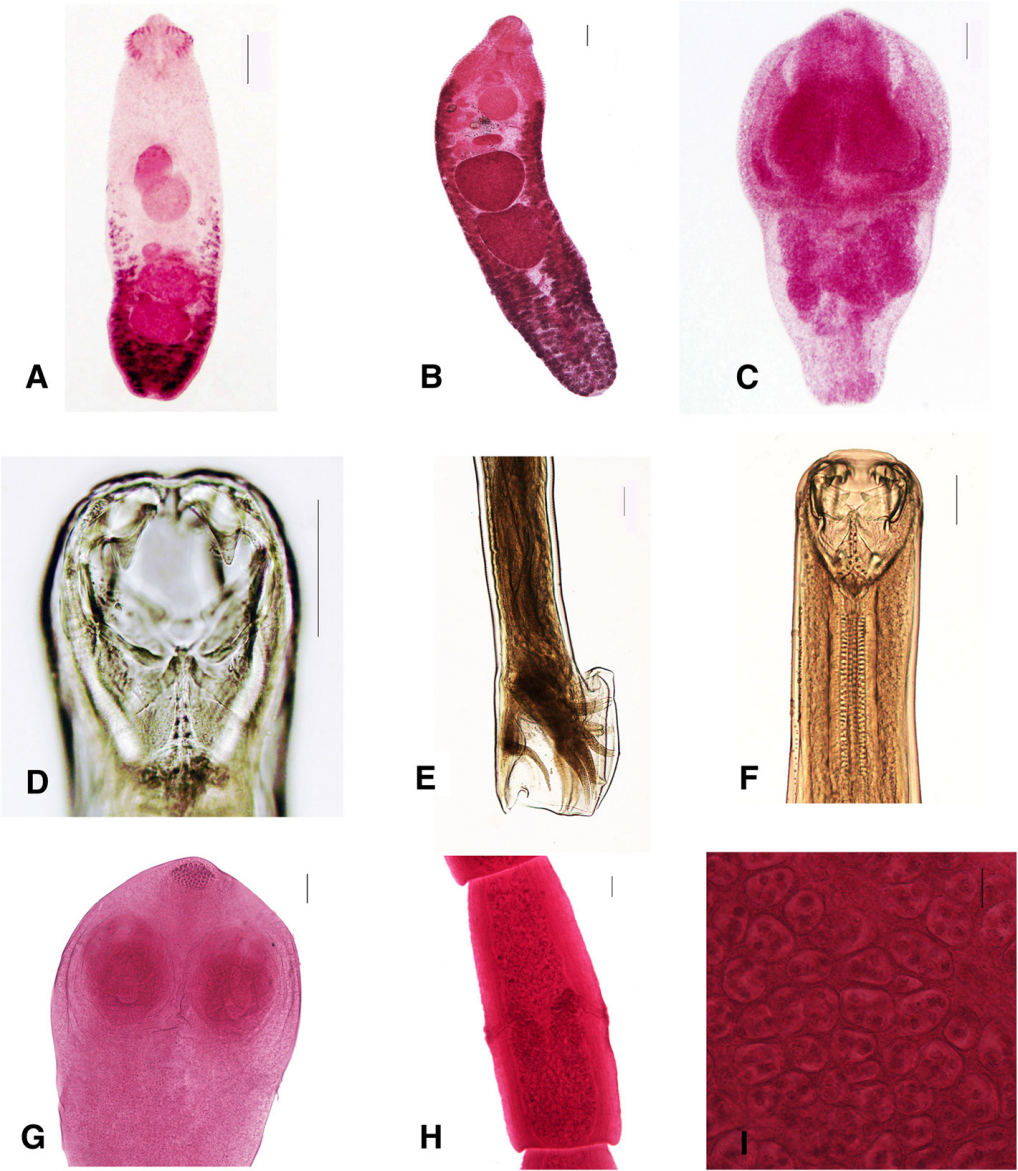
Some of the gastrointestinal helminths identified in feral dogs and cats. **a** *Echinochasmus liliputanus* from a dog; **b** *Echinochasmus perfoliatus* from a cat. The body surface is covered with spines from the collar to the anterior testis; (**c**) *Pharyngostomum cordatum* from a cat; **d** Buccal capsule of *Ancylostoma ceylanicum* from a cat. The anterior edge of the buccal capsule is armed with a pair of large hook-like teeth; **e** Lateral view of male bursa of *Ancylostoma ceylanicum* from a cat; **f** *Ancylostoma caninum* from a dog. The wide buccal capsule bears three pairs of ventral teeth; **g** Rostellum of *Dipylidium caninum* from a dog. Identification characteristics include a rostellum armed with several alternating rows of thorn-shaped hooks, uterine capsules with several eggs, a vagina posterior to the cirrus-sac; **h** Mature proglottid of *Dipylidium caninum* from a dog; **i** Egg capsules of gravid proglottid of *Dipylidium caninum* from a dog. Scale bar = 100 μm

Gastrointestinal helminths were found in all the dogs. Eleven species of helminths were identified, including 5trematodes, 3 cestodes, and 3 nematodes (Fig. 1 and Table 1). The predominant parasite was the cestode *Dipylidium caninum*, detected in 72.5 % of the examined dogs. The overall infection rate of dogs with more than one helminth species was 77.5 %.

**Table 1:** Gastrointestinal helminths identified in feral dogs (*n* = 40) in Guangxi, China

Parasite species	Number of infected dogs (prevalence)	Number of parasites per dog (mean)
Trematodes		
*Clonorchis sinensis*	11 (27.5 %)	1–41 (6.1)
*Echinochasmus perfoliatus*	4 (10.0 %)	not done
*Echinochasmus liliputanus*	4 (10.0 %)	not done
*Metagonimus yokogawai*	2 (5.0 %)	6–18 (12.0)
*Haplorchis taichui*	5 (12.5 %)	3–40 (15.2)
Cestodes		
*Dipylidium caninum*	29 (72.5 %)	1–58 (19.2)
*Taenia taeniaeformis*	2 (5.0 %)	15–30 (22.5)
*Spirometra erinaceieuropaei*	4 (10.0 %)	1–4 (2.3)
Nematodes		
*Ancylostoma ceylanicum*	7 (17.5 %)	1–15 (8.6)
*Ancylostoma caninum*	4 (10.0 %)	1–10 (6.0)
*Toxocara canis*	12 (30.0 %)	1–12 (3.4)

Gastrointestinal helminths were found in 37 out of 39 cats. Twelve species of helminths were identified, including 6 trematodes, 3 cestodes and 3 nematodes (Fig. 1 and Table 2). The Predominant parasites were the cestode *Dipylidium caninum* (in 72.5 % of the cats), the liver fluke *Clonorchis sinensis* (33.9 %) and the cestode *Spirometra erinaceieuropaei* (33.3 %). The overall infection rate of cats with more than one helminth species was 73.0 %.

**Table 2:** Gastrointestinal helminths identified in feral cats (*n* = 39) in Guangxi, China

Parasite species	Number of infected cats (prevalence)	Number of parasites per cat (mean)
Trematodes		
*Clonorchis sinensis*	14 (35.9 %)	1–1153 (209.1)
*Echinochasmus perfoliatus*	10 (25.6 %)	2–638 (67.6)
*Metagonimus yokogawai*	5 (12.8 %)	8–135 (40.8)
*Haplorchis taichui*	1 (2.6 %)	33
*Haplorchis pumilio*	2 (5.1 %)	65–148 (139)
*Pharyngostomum cordatum*	8 (20.5 %)	21–718 (239.8)
Cestodes		
*Dipylidium caninum*	15 (38.5 %)	1–824 (162.5)
*Taenia taeniaeformis*	6 (15.4 %)	1–9 (3.2)
*Spirometra erinaceieuropaei*	13 (33.3 %)	1–12 (2.6)
Nematodes		
*Ancylostoma ceylanicum*	11 (28.2 %)	1–32 (7.7)
*Toxocara cati*	2 (5.1 %)	2
*Toxascaris leonina*	3 (7.7 %)	2

Among the 14 species of helminths identified in the present survey, *Echinochasmus liliputanus*, *Pharyngostomum cordatum* and *Ancylostoma ceylanicum* were reported for the first time in Guangxi province. The main characteristics concerning the morphology and biology of these parasites are presented in Table 3.

**Table 3:** Main characteristics of the helminth species, which were detected for the first time in dogs and cats from Guangxi, China

Parasite species	Morphological characteristics	Definitive (DH) and intermediate (IH) hosts
*Echinochasmus liliputanus* (Trematode, Echinostomatidae)	1.52–2.06 × 0.46–0.56 mm	DH: dogs, cats, humans
	A row of 24 collar spines is present; the vitellaria are distributed from the posterior end of acetabulum to terminal; the body surface is covered with spines from the collar to the posterior testis (Fig. 1a)	
		IH: snails, bivalves, crustaceans, fishes, and amphibians [14]
*Pharyngostomum cordatum* (Trematode, Diplostomidae)	1.40–2.10 × 1.02–1.52 mm	DH: cats, lions [16]
	Indistinctly bipartite body, a huge holdfast organ, cordiform, and irregular oval testes (Fig. 1c)	IH: snails, tadpoles
		Reservoir host: toad, snakes, tortoises and shrews [17]
*Ancylostoma ceylanicum* (Nematode, Ancylostomatidae)	Male: 5.26–6.50 × 0.23–0.26 cm	Dog, cats, humans [3]
	Female: 5.79–6.70 × 0.25–0.31 cm	
	The anterior edge of the buccal capsule is armed with a pair of large hook-like teeth (Fig. 1d & e)	

Nematodes of the genus *Strongyloides* and cestodes of the genus *Echinococcus* were not detected in the present study.

In China, only little information is available about the prevalence of gastrointestinal parasites in dogs and cats. Andrews [7] first reported the presence of helminths in dogs and cats in Shanghai. Wang et al. [8] and Dai et al. [9] identified helminths in 178 dogs from Heilongjiang province, and in 438 dogs from Hunan province, respectively. The present study demonstrated for the first time that gastrointestinal helminths are common in feral carnivores in Guangxi province. In dogs, the infection rate was 100 %, the same value as that reported by Wang et al. [8] in Heilongjiang and Dai et al. [9] in Hunan. In feral cats, the infection rate was 94.9 %, a value similar to that reported by Yang [10] in Sichuan (95 %) and Wang et al. [11] in Guizhou (88.6 %).

The most important result of the present study is that most of the detected helminths can infect humans. In both dogs and cats, the most frequently identified parasite was the cestode *Dipylidium caninum*, suggesting a high density of flea intermediate hosts in feral carnivores. Humans, normally children, acquire the infection by accidentally ingesting infected fleas [12].

The prevalence of the cestode *Spirometra erinaceieuropaei* infection in cats (33.3 %) was higher than in dogs (10.0 %). This may be due to the fact that cats hunt amphibians, reptiles, and small mammals (which represent intermediate or paratenic host for *S. erinaceieuropaei*) more frequently than dogs do. During 1927–2009, more than 1000 cases in humans in 25 Chinese provinces were reported; most cases were in southern China, where human infections were mainly acquired by eating raw or insufficiently cooked meat of frogs and snakes or by placing frog or snake flesh on open wounds for treatment of skin ulcers or on eyes to treat inflammation [13, 14]. The high infection rate in definitive hosts probably maintains a high level of contamination in frogs and snakes and consequently a high risk of sparganosis in local residents who have the habit of eating meat of frogs and snakes and some superstitious beliefs in medical properties of raw frog or snake meat [14].

The trematode species *Echinochasmus liliputanus* was found only in dogs with an infection rate of 10.0 %. This parasite can infect animals and humans as definitive hosts via both metacercariae and cercariae. Through 2002, more than 2500 human cases have been reported in Anhui province, China. Drinking unboiled pond water containing cercariae is the main route of human infection [15–17].

*Clonorchis sinensis*, *Echinochasmus perfoliatus*, *Metagonimus yokogawai*, *Haplorchis taichui* and *H. pumilio* are liver or intestinal flukes, which can infect both carnivores and humans as definitive hosts. Humans become infected through ingestion of raw or undercooked freshwater fish or shrimp infected with metacercariae [17, 18]. Since raw fish is a popular dish in Guangxi, the high prevalence and intensity of liver and intestinal flukes in feral carnivores is of real concern. According to a recent survey based on coproscopic examinations, 59.6 % (428/718) of Guangxi inhabitants are infected by *Clonorchis sinensis* and intestinal flukes [19].

**Fig. 2:**
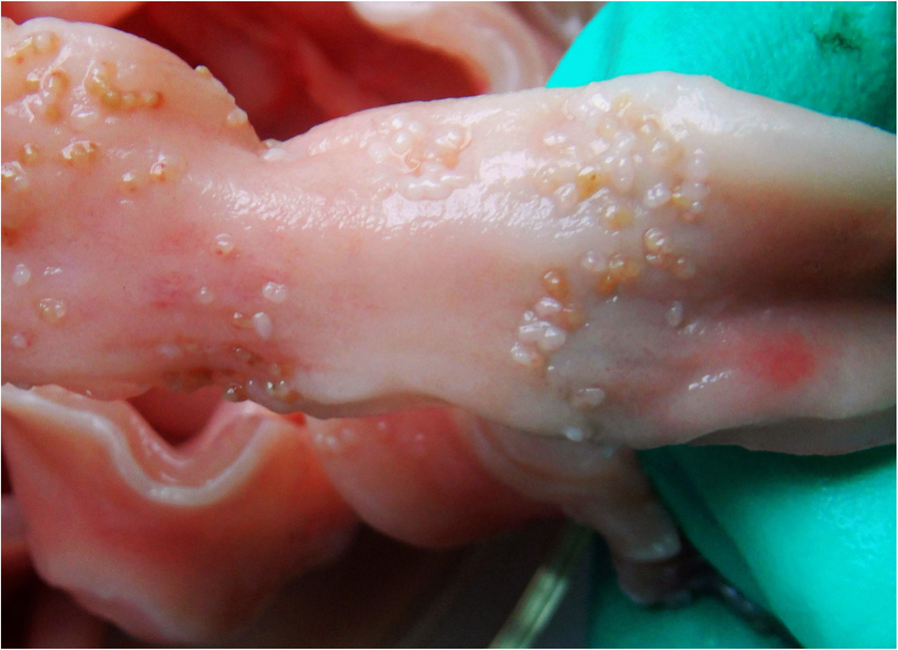
Adults of the trematode species *Pharyngostomum cordatum* on the pharynx of a cat

The trematode species *Pharyngostomum cordatum* is a feline parasite, mainly distributed in Southern China (Fig. 2) [20]. In the present survey the frequency in feral cats (20.5 %) was greater than that reported by Yang [10] in Sichuan (15.0 %). According to Shin [21], humans are likely to be infected with *P. cordatum* as paratenic host. However, no human infection has been reported so far.

Both hookworms *Ancylostoma caninum* and *A. ceylanicum* can infect humans [3]. In the present study, the predominant species was *A. ceylanicum*, with an infection rate of 17.5 % in dogs and 28.2 % in cats, and it was the only hookworm species found in the examined cats. Human infections with *A. ceylanicum* were reported in Fujian where the high prevalence of hookworm infections in dogs and cats was identified as a significant risk factor for human contamination [22]. According to Xu et al. (2005), the species *Necator americanus* and *A. duodenale* are found to be the most prevalent hookworms distributed in provinces of southern China (including Guangxi), with a mean prevalence of 6.12 % nationwide [23]. Infected people mainly live in less developed rural areas, where dogs and cats may roam freely and farmers often walk barefoot [24].

*Toxocara canis* infection rate (30 %) reported in the present study in dogs was lower than that previously reported from Heilongjiang (36.5 %) and Hunan (45.2 %), respectively [8, 9]. In cats, infection rates with *Toxocara cati* (5.1 %) and *Toxascaris leonina* (7.7 %) were relatively low in comparison to previous surveys [10, 11]. Toxocarosis is considered as one of the most common parasitic zoonoses in the world and a high incidence has been reported in developing countries. However a few investigations have been made in China and there is only one report indicating that contact with infected dogs is the risk factor for human toxocarosis in China [25]. With regard to *Toxocara* in cats, Li et al. [26] mentioned the presence of a new *Toxocara* species (*T. malaysiensis*) in Guangzhou, China. This parasite seems to be remarkably different from *T. canis*, *T. cati* and *T. leonina* of dogs and cats by molecular characterization [26]. However, the role of *T. malaysiensis* as a zoonotic parasite has yet to be confirmed.

## Conclusions

The present study clearly demonstrated that major zoonotic gastrointestinal helminths are present in feral dogs and cats in Guangxi, China. They may represent a significant risk for public health and appropriate measures should be taken to regulate the populations of feral carnivores and to promote deworming programs for dogs and cats. Additional preventive measures include protection of aquaculture systems from contamination with feces from dogs and cats and development of detection methods for foodborne parasitic infection at the processing, distribution and buying stages.

## References

[1] Deplazes P, van Knapen F, Schweiger A, Overgaauw PA (2011). Role of pet dogs and cats in the transmission of helminthic zoonoses in Europe, with a focus on echinococcosis and toxocarosis. Vet Parasitol.

[2] Chen J, Xu MJ, Zhou DH, Song HQ, Wang CR, Zhu XQ (2012). Canine and feline parasitic zoonoses in China. Parasit Vectors.

[3] Bowman DD, Montgomery SP, Zajac AM, Eberhard ML, Kazacos KR (2010). Hookworms of dogs and cats as agents of cutaneous larva migrans. Trends Parasitol.

[4] Yamaguti S (1971). Synopsis of digenetic trematodes of vertebrates.

[5] Wu SQ (2001). Fauna sinica, nematoda, rhabditida: Strongylata (I).

[6] Khalil LF, Jones A, Bray RA. Keys to the cestode parasites of vertebrates. CAB International, Wallingford, UK, 1994.

[7] Andrews MN (1937). The helminth parasites of dogs and cats in Shanghai, China. J Helminthol.

[8] Wang CR, Qiu JH, Zhao JP, Xu LM, Yu WC, Zhu XQ (2006). Prevalence of helminths in adult dogs in Heilongjiang Province, the People’s Republic of China. Parasitol Res.

[9] Dai RS, Li ZY, Li F, Liu DX, Liu W, Liu GH, He SW, Tan MY, Lin RQ, Liu Y, Zhu XQ (2009). Severe infection of adult dogs with helminths in Hunan Province, China poses significant public health concerns. Vet Parasitol.

[10] Yang MF (1987). Investigation of felid parasites in Sichuan. Sichuan J Agr Sci.

[11] Wang DD, Liu XM, Han X (1995). Prevalence of helminths of dogs and cats in Guizhou province. Chin J Vet Science Technol.

[12] Chappell CL, Penn HM (1990). *Dipylidium caninum*, an underrecognized infection in infants and children. Pediatr Infect Dis J.

[13] Wang FM, Zhou LH, Gong SP, Deng YZ, Zou JJ, Wu J, Hou FH (2011). Severe infection of wild-caught snakes with *Spirometra erinaceieuropaei* from food markets in Guangzhou, China involves a risk for zoonotic sparganosis. J Parasitol.

[14] Li MW, Song HQ, Li C, Lin HY, Xie WT, Lin RQ, Zhu XQ (2011). Sparganosis in mainland China. Int J Infect Dis.

[15] Xiao X, Lu DB, Wang TP, Gao JF, Wu WD (1994). Epidemiological studies on *Echinochasmus liliputanus* infection I. Parasite infection and distribution in final hosts. Chin J Parasitol Parasit Dis.

[16] Xiao X, Wang T, Zheng X, Shen G, Tian Z (2005). *In vivo* and *in vitro* encystment of *Echinochasmus liliputanus* cercariae and biological activity of the metacercariae. J Parasitol.

[17] Sohn WM, Eom KS, Min DY, Rim HJ, Hoang EH, Yang Y, Li X (2009). Fishborne trematode metacercariae in freshwater fish from Guangxi Zhuang Autonomous Region, China. Korean J Parasitol.

[18] Graczyk TK, Fried B (2007). Human waterborne trematode and protozoan infections. Adv Parasitol.

[19] Jeon HK, Lee D, Park H, Min DY, Rim HJ, Zhang HM, Yang YC, Li XM, Eom KS (2012). Human infections with liver and minute intestinal flukes in Guangxi, China: analysis by DNA sequencing, ultrasonography, and immunoaffinity chromatography. Kor J Parasitol.

[20] Wallace FG (1939). The life cycle of *Pharyngostomum cordatum* (Diesing) Ciurea (Trematoda: Alariidae). Trans Am Micro Soc.

[21] Shin EH, Chai JY, Lee SH (2001). Extraintestinal migration of *Pharyngostomum cordatum* metacercariae in experimental rodents. J Helminthol.

[22] Chen QX, Lin XM, Yang CC (1981). Studies on the biology and epidemiology of *Ancylostoma ceylanicum* infection from man and animal in Fujian, China. J Xiamen Univ.

[23] Xu LQ, Chen YD, Sun FH, Cai L, Fang RY, Wang LP, Liu X, Feng Y, Li H (2005). A national survey on current status of the important parasitic diseases in human population. Chin J Parasitol Parasit Dis.

[24] Xie H, Tian HC, Wang XG, Liu CH, Zhen DF, Tan K, Liu WL, Hu M, Tang ZJ, Chen YL, Zhang FN (2011). Surveillance of geohelminthiasis in national surveillance area in Sichuan, 2006–2009. J Prev Med Infect.

[25] Li XR, Dong YX, Duan YH, Zeng GX, Wang FY, Wang YB (2009). A reported case of *Toxocara canis*. Livestock Poult Indust.

[26] Li MW, Zhu XQ, Gasser RB, Lin RQ, Sani RA, Lun ZR, Jacobs DE (2006). The occurrence of *Toxocara malaysiensis* in cats in China, confirmed by sequence-based analyses of ribosomal DNA. Parasitol Res.

